# Assessment of Milk and Beverage Intake Trends During Preschool Age and Modeling the Nutritional Impact of Replacing Nondairy Caloric Beverages with Milk

**DOI:** 10.1016/j.cdnut.2024.104436

**Published:** 2024-08-08

**Authors:** Kristin Ricklefs-Johnson, Matthew A Pikosky, Christopher J Cifelli, Kristin Fulgoni, Victor L Fulgoni, Sanjiv Agarwal

**Affiliations:** 1 National Dairy Council, Rosemont, IL, United States; 2 Nutrition Impact, LLC, Battle Creek, MI, United States; 3 NutriScience, LLC, East Norriton, PA, United States

**Keywords:** beverages, calcium, children, magnesium, milk, NHANES, protein, SSB, vitamin D

## Abstract

**Background:**

Milk provides essential crucial public health nutrients, including 3–4 nutrients of public health concern, yet dairy consumption has declined over time, leading most Americans to fall short of meeting Dietary Guidelines recommendations.

**Objectives:**

To investigate milk and beverage consumption trends in preschool-age children, along with nutrient intakes from beverages, and to analyze the potential impact of replacing nondairy beverages with milk through isocaloric substitution.

**Methods:**

Data from the National Health and Nutrition Examination Survey 2001–2018 for children aged 1–5 y (*n* = 4696) were used, and milk and other beverages intakes were estimated from the first 24-h in-person dietary recall. Nutrient intakes were determined using the United States Department of Agriculture’s food and nutrient database for dietary studies. Changes in nutrient intakes of children aged 2–5 y were modeled assuming isocaloric substitution with milk of all nondairy beverages consumed during lunch and dinner combined. Sample-weighted analyses were performed using SAS 9.4, and significance was set at *P* < 0.01.

**Results:**

With the increasing age of children, the intake of milk decreased, whereas the intake of energy, caloric beverages excluding milk, and sugar-sweetened beverages increased. Daily intakes of energy, protein, fat, saturated fatty acids (SFA), calcium, magnesium, potassium, sodium, vitamin A, folate, vitamin B-12, and vitamin D from caloric beverages including milk decreased with age, whereas the daily intake of fiber and added sugar increased with age. With the isocaloric replacement of nondairy caloric beverages with milk at lunch and dinner among children aged 2–5 y, intake of protein, fat, SFAs, calcium, magnesium, potassium, sodium, vitamin A, folate, vitamin B-12, and vitamin D increased, whereas for intake of carbohydrate, fiber, total sugar, and added sugar decreased.

**Conclusions:**

The current findings indicate that increased efforts are needed to reverse the decrease in milk intake over time and as preschool children age and provide additional evidence to support specific dietary recommendations for milk.

## Introduction

Early childhood is a phase marked by swift growth and development, accompanied by distinctive nutritional requirements crucial for fostering optimal physical and cognitive growth [[1], [2], [3], [4], [5]]. It is also a transition period from an exclusive breast milk or infant formula diet to a more diverse diet comprised of whole foods and beverages, with children having increased autonomy regarding what they eat. Food preferences, behaviors, and dietary patterns developed during these early life stages often serve as a foundational basis for future food/beverage preferences, which may influence lifetime nutritional status [[6], [7], [8], [9]]. Beverages make a significant contribution to energy and essential nutrients in the diets of children [10,11], and therefore, healthy beverage intake is critical in early childhood. Many authoritative bodies, including the American Academy of Pediatrics and the Dietary Guidelines for Americans (DGA) 2020–2025, have provided beverage intake recommendations for children [[12], [13], [14], [15], [16]]. The recommended beverages for children under age 5 include breast milk, plain cow milk, water, and 100% fruit juice. Plant milk and nondairy beverages, including sugar-sweetened beverages (SSBs), are not recommended except for fortified soy beverages, which can be included when medically indicated or to meet specific dietary preferences [[12], [13], [14], [15], [16]]. Despite these universal recommendations, data suggest that nearly 61% of children aged 2–19 y consume SSB on a given day [17]. Nearly half (46%) of children aged 2–4 y consumed SSB on the day of the survey in the feeding infants and toddlers study 2016 study [18], and 57% of children aged 1–5 y consumed SSB at least once a week in National Survey of Children’s Health, United States, 2021 [19]. The overconsumption of SSBs among children has been linked to adverse health effects in some studies [[20], [21], [22], [23]].

Cow milk is a good source of key essential nutrients, including high-quality protein, calcium, phosphorus, iodine, potassium, selenium, zinc, vitamin A, vitamin D, riboflavin (vitamin B_2_), niacin (vitamin B_3_), pantothenic acid (vitamin B_5_) and cobalamin (vitamin B-12) [24,25]. Dairy products, including milk, make a significant contribution to nutrient intakes among Americans, and research suggests that children 2 y of age and older meeting dairy recommendations are less likely to be below recommended intakes (Estimated average requirements and adequate intakes) for several essential nutrients including protein, calcium, magnesium, phosphorus, selenium, potassium, riboflavin, vitamin A, vitamin B-12, vitamin D and choline [26]. Milk was identified as the leading food source ensuring adequacy for calcium, potassium, and vitamin D, providing ∼53% of vitamin D, 33% of calcium, and 19% potassium to daily intakes of American children aged 2–5 y [27].

Data suggest that 85–90% of the American population, including over 40% of children aged 2+ y, do not consume the recommended amount of milk [28]. Inadequate milk intake among children makes it difficult for them to meet nutrient recommendations, potentially resulting in adverse effects on growth, cognitive development, bone health, and weight gain in childhood [29]. Additionally, the increasing intakes of soft drinks and fruit-flavored drinks, which can contain large amounts of added sugar and calories and are typically poor in nutrients, appear to be displacing milk, which is concerning [12]. To date, there are only a limited number of studies that have focused on beverage consumption patterns and their contribution to nutrient intake during early childhood. Therefore, the objective of this study was to investigate current trends in beverage intake as well as nutrient intake status in preschool-age United States children (age 1–5 y)using the NHANES data set, a nationally representative, continuous survey of the United States population [30].

## Methods

### Subjects

Data from What We Eat In America, a nutritional component of NHANES, was used to assess the dietary intakes of children aged 1–5 y. NHANES is a large continuous dietary survey of a nationally representative sample of the United States population, and the present analysis combined 9 NHANES datasets over 2 decades (NHANES 2001–2002, 2003–2004, 2005–2006, 2007–2008, 2009–2010, 2011–2012, 2013–2014, 2015–2016, and 2017–2018) [30]. The combined sample included 9099 children aged 1–5 y after excluding those with incomplete or unreliable dietary recalls. Written informed consent was obtained from all parents/caretakers of the children’s participants, and the research ethics review board at the National Center for Health Statistics (NCHS) approved the survey protocol. This study was the analysis of secondary data without any personal identifiers and, therefore, did not require further institutional review.

### Estimation of intake

The first 24-h in-person dietary recall data of children and/or provided by parents/caretakers was used to estimate milk and beverage intake. A complete description of NHANES data collection protocols is provided elsewhere [30,31]. Milk and other beverages were defined by using the following USDA food codes: milk - USDA subgroup 10, flavored milk - USDA subgroup 12, milk substitutes - USDA category 1404, fruit juice - USDA subgroup 70, SSB - USDA subgroup 72, soft drinks - USDA category 7202, fruit drinks - USDA category 7204, coffee and tea - USDA subgroup 73, caloric beverages excluding milk (CB) - USDA main group 8, and subgroups not in 71, 77, 78, and caloric beverages including milk (CB + M) - also included milk, flavored milk and milk substitutes [32]. Water (tap or bottled) was not considered in this study as it provides no calories and typically only limited nutrients. Energy and nutrient intake were determined using the respective Food & Nutrient Database for Dietary Studies for each NHANES cycle [32].

### Nutrient modeling

Nutrient modeling was performed by *1*) adding 1 cup (∼225 g) equivalent serving of milk to the diet of each subject and *2*) an isocaloric substitution of all nonmilk caloric beverages (SSB, milk substitutes (including plant-based), or all beverages in What We Eat In America beverage subgroup) consumed during lunch and dinner meals with milk. The nutrient profile of NHANES food code 11100000 milk, not further specified, which is a combination of various fat concentrations of milk (nfs), was used in both modeling scenarios (Supplemental Table 1). Nutrient modeling was only performed for children aged 2–5 y due to low amounts of consumption of other nonmilk caloric beverages in children aged 1 y.

### Statistical analyses

All analyses were performed using SAS 9.4 (SAS Institute) using PROC SURVEYMEANS after adjusting the data for the complex sampling design of NHANES, using appropriate survey weights, strata, and primary sampling units. The recommended population ratio method was used to assess the nutritional contribution of caloric beverages. Separate analyses were conducted for age groups (1, 2, 3, 4, and 5 y). Regression analyses were used to assess changes in intake across age groups using PROC SURVEYREG after adjusting data for gender, ethnicity, and poverty-income ratio (PIR), with the regression coefficient for age representing the change across ages. These covariates were chosen because they are known to be associated with beverage choices. Paired t-tests were used to assess changes in intake for modeling of replacement of caloric beverages at meals.

## Results

### Demographics

Approximately 50% of children were male, and across age groups, 53–57% were non-Hispanic White, followed by Mexican Americans (16–17%) and non-Hispanic Black (13–14%). Approximately 50% of children were in the higher household income group (PIR > 1.85) category, and 37–41% of children were in the lowest household income group (PIR < 1.35) category. Approximately 70% of children aged 2–5 y reported having vigorous physical activity (physical activity data for children aged 1 y was not available), and 68–75% of children across all age groups were of normal weight (Table 1).

**TABLE 1:** Demographics of children aged 1–5 y, NHANES 2001–2018.

Variable	Age 1 y	Age 2 y	Age 3 y	Age 4 y	Age 5 y
*N*	2169	2325	1539	1590	1476
Male (%)	51.6 ± 1.5	49.1 ± 1.4	51.5 ± 1.9	50.7 ± 1.8	50.4 ± 1.8
Ethnicity					
Mexican American (%)	16.6 ± 1.3	16.5 ± 1.3	15.9 ± 1.6	16.1 ± 1.3	16.3 ± 1.5
Other Hispanic (%)	8.16 ± 0.83	7.32 ± 0.81	7.78 ± 0.86	5.93 ± 0.76	6.68 ± 0.77
Non-Hispanic White (%)	54.2 ± 2.0	54.3 ± 2.0	52.6 ± 2.3	53.6 ± 2.2	56.8 ± 2.1
Non-Hispanic Black (%)	13.4 ± 1.0	13.7 ± 1.1	14.3 ± 1.2	13.9 ± 1.1	13.2 ± 1.2
Other (%)	7.60 ± 0.76	8.22 ± 0.67	9.33 ± 1.10	10.5 ± 1.0	7.04 ± 0.82
Poverty-income-ratio					
<1.35 (%)	40.7 ± 1.4	39.9 ± 1.5	37.0 ± 1.9	39.7 ± 1.8	38.1 ± 1.9
1.35 –≤1.85 (%)	10.6 ± 1.0	10.8 ± 1.0	12.1 ± 1.1	10.3 ± 1.2	11.1 ± 1.3
>1.85 (%)	48.7 ± 1.7	49.3 ± 1.7	50.8 ± 2.1	50.0 ± 2.0	50.8 ± 2.0
Physical activity					
Sedentary (%)		14.2 ± 1.0	12.3 ± 1.1	12.3 ± 1.1	12.1 ± 1.3
Moderate (%)		13.0 ± 1.2	16.0 ± 1.6	15.8 ± 1.3	19.9 ± 1.6
Vigorous (%)		72.7 ± 1.4	71.7 ± 1.9	71.9 ± 1.6	68.0 ± 1.7
Weight status					
Underweight (%)	3.62 ± 0.52	2.94 ± 0.44	3.53 ± 0.73	2.84 ± 0.57	2.82 ± 0.60
Normal weight (%)	74.9 ± 1.3	74.1 ± 1.2	72.7 ± 1.7	70.4 ± 1.6	68.4 ± 1.6
Overweight (%)	13.0 ± 1.1	14.9 ± 1.2	14.4 ± 1.5	12.8 ± 1.1	13.2 ± 1.2
Obese (%)	8.5 ± 0.8	8.0 ± 0.8	9.4 ± 1.0	14.0 ± 1.2	15.6 ± 1.3

Abbreviations: NHANES, National Health and Nutrition Examination Survey; SEM, standard error of the mean.

Data presented as mean ± SEM. The poverty-income-ratio is a measure of household income relative to the federal poverty level.

### Intake of beverages

Daily intake of energy increased, whereas intakes of milk, milk substitutes, CB + M and 100% fruit juice decreased with higher ages, and the changes in intake were +108 kcal/d/y of age (regression coefficient for linear trend:β_linear trend_; *P* < 0.0001) for energy, –60.4 g/d/y of age (*P* < 0.0001) for milk, –3.01 g/d/y of age (*P* = 0.0003) for milk substitutes, –23.9 g/d/y of age (*P* < 0.0001) CB + M and –10.2 g/d/y of age (*P* = 0.0001) for 100% fruit juice. Daily intakes of other beverages increased with age of children: flavored milk (β_linear trend_ = +14.8 g/d/y of age, *P* < 0.0001), CB (β_linear trend_ = +24.7 g/d/y of age, *P* < 0.0001), fruit drinks (β_linear trend_ = +16.0 g/d/y of age, *P* < 0.0001), soft drinks (β_linear trend_ = +16.1 g/d/y of age, *P* < 0.0001), and SSBs (β_linear trend_ = +31.8 g/d/y of age, *P* < 0.0001) (Table 2).

**TABLE 2:** Mean intake of beverages among children aged 1–5 y, gender-combined data NHANES 2001–2018.

Nutrient	Age 1 y	Age 2 y	Age 3 y	Age 4 y	Age 5 y	Regression coefficient	*P*_-linear trend_
Energy (kcal)	1289 ± 17	1422 ± 13	1518 ± 19	1594 ± 18	1742 ± 21	108 ± 6	<0.0001
Milk (g)	487 ± 12	363 ± 10	277 ± 10	256 ± 10	237 ± 10	–60 ± 3	<0.0001
Flavored milk (g)	17.5 ± 3.0	50.1 ± 4.8	57.4 ± 5.5	65.3 ± 5.5	84.7 ± 7.9	14.8 ± 1.8	<0.0001
Milk substitutes (g)	18.3 ± 3.3	8.95 ± 2.10	9.93 ± 2.24	6.85 ± 1.89	4.12 ± 1.65	–3.01 ± 0.82	0.0003
Caloric beverages, including milk (g)	795 ± 16	757 ± 13	676 ± 16	672 ± 17	718 ± 19	–24 ± 5	<0.0001
Caloric beverages, excluding milk (g)	272 ± 10	335 ± 10	331 ± 13	343 ± 13	393 ± 16	25 ± 4	<0.0001
100% Juice (g)	153 ± 7	169 ± 8	156 ± 7	139 ± 10	118 ± 8	–10 ± 2	0.0001
Fruit drinks (g)	72.5 ± 5.4	105 ± 6	94.0 ± 6.2	110 ± 7	151 ± 11	16.0 ± 2.6	<0.0001
Soft drinks (g)	16.5 ± 2.9	30.6 ± 3.0	45.7 ± 3.8	61.0 ± 4.6	81.7 ± 6.2	16.1 ± 1.5	<0.0001
SSB (g)	103 ± 7	148 ± 7	148 ± 8	184 ± 9	245 ± 13	32 ± 3	<0.0001
Coffee, tea (g)	16.0 ± 3.4	17.7 ± 2.3	28.0 ± 5.0	20.4 ± 2.8	29.9 ± 5.3	3.07 ± 1.25	0.0150

Abbreviations: NHANES, National Health and Nutrition Examination Survey; SEM, standard error of the mean; SSB, sugar-sweetened beverage.

Data presented as least square mean ± SEM after adjusting for gender, ethnicity, and poverty-income-ratio level. The regression coefficient represents the change across ages 1–5 y and indicates the change per 1 y of age.

### Changes in nutrient intake from CB + M

Daily intake of nutrients from CB + M decreased with age and percentage decreases between ages 1 y and 5 y were: energy (–17%), protein (–35%), fat (–52%), MUFA (–51%), PUFA (–58%), SFA (–51%), calcium (–32%), magnesium (–24%), potassium (–26%), sodium (–24%), vitamin A (–27%), folate (–19%), vitamin B-12 (–39%) and vitamin D (–39%); and the daily intake of fiber (55%) and added sugar (162%) increased (Table 3).

**TABLE 3:** Mean intake of nutrients from caloric beverages, including milk among children aged 1–5 y, gender-combined data NHANES 2001–2018.

Nutrient	Age 1 y	Age 2 y	Age 3 y	Age 4 y	Age 5 y	Regression coefficient	*P*-_linear trend_
Energy (kcal)	421 ± 9	379 ± 7	334 ± 8	330 ± 9	348 ± 10	–19 ± 3	<0.0001
Carbohydrate (g)	55.3 ± 1.4	59.3 ± 1.3	55.7 ± 1.5	56.4 ± 1.6	61.2 ± 1.8	0.8 ± 4	0.0624
Dietary fiber (g)	0.48 ± 0.03	0.66 ± 0.04	0.64 ± 0.03	0.66 ± 0.04	0.75 ± 0.05	0.05 ± 0.01	0.0002
Total sugars (g)	53.5 ± 1.3	55.6 ± 1.1	52.1 ± 1.5	52.4 ± 1.4	57.1 ± 1.7	0.4 ± 0.4	0.3722
Added sugars (tsp eq)	2.48 ± 0.15	3.79 ± 0.16	4.24 ± 0.21	4.92 ± 0.22	6.49 ± 0.32	0.91 ± 0.07	<0.0001
Protein (g)	17.1 ± 0.4	14.2 ± 0.3	11.6 ± 0.4	11.2 ± 0.4	11.1 ± 0.4	–1.5 ± 0.1	<0.0001
Total fat (g)	15.0 ± 0.3	9.88 ± 0.26	7.63 ± 0.26	7.18 ± 0.26	7.18 ± 0.32	–1.81 ± 0.09	<0.0001
Total MUFA (g)	3.76 ± 0.09	2.53 ± 0.07	1.96 ± 0.07	1.84 ± 0.07	1.85 ± 0.08	–0.45 ± 0.02	<0.0001
Total PUFA (g)	0.97 ± 0.03	0.58 ± 0.02	0.47 ± 0.02	0.43 ± 0.02	0.41 ± 0.02	–0.13 ± 0.01	<0.0001
Total SFA (g)	8.56 ± 0.20	5.76 ± 0.15	4.41 ± 0.16	4.18 ± 0.15	4.22 ± 0.20	–1.01 ± 0.05	<0.0001
Calcium (mg)	644 ± 14	552 ± 12	462 ± 14	450 ± 17	436 ± 15	–52 ± 4	<0.0001
Magnesium (mg)	69.1 ± 1.5	64.4 ± 1.3	56 ± 2	54.1 ± 1.7	52.8 ± 1.6	–4.3 ± 0.4	<0.0001
Potassium (mg)	958 ± 21	872 ± 17	756 ± 19	719 ± 23	712 ± 23	–65 ± 6	<0.0001
Sodium (mg)	252 ± 6	219 ± 5	184 ± 5	180 ± 5	190 ± 6	–16 ± 2	<0.0001
Vitamin A, RE (μg)	238 ± 7	219 ± 6	181 ± 6	180 ± 7	175 ± 8	–17 ± 2	<0.0001
Folate, DFE (μg)	34.3 ± 1.0	32.1 ± 0.9	29.8 ± 1.8	29.1 ± 1.8	27.7 ± 1.3	–1.6 ± 0.4	<0.0001
Vitamin B-12 (μg)	2.45 ± 0.05	2.03 ± 0.05	1.63 ± 0.05	1.56 ± 0.06	1.49 ± 0.06	–0.24 ± 0.02	<0.0001
Vitamin D (μg)	6.60 ± 0.15	5.22 ± 0.13	4.23 ± 0.12	4.12 ± 0.14	4.05 ± 0.15	–0.61 ± 0.04	<0.0001

Abbreviations: DFE, dietary folate equivalents; MUFA, monounsaturated fatty acid; NHANES, National Health and Nutrition Examination Survey; PUFA, polyunsaturated fatty acid; RE, retinol equivalent; SEM, standard error of the mean; SFA, saturated fatty acid.

Data presented as least square mean ± SEM after adjusting for gender, ethnicity, and poverty-income-ratio level. The regression coefficient represents the change across ages 1–5 y and indicates the change per 1 y of age.

### Modeling nutrient intake with the addition of milk

With the addition of 1 cup of milk (NHANES food code 11100000 milk, nfs) to the diets of children aged 2–5 y, intake of several nutrients increased across age groups including calcium (29–31%), magnesium (13–14%), potassium (17–18%), protein (13–15%), SFAs (13–15%), total sugars (10–12%), and vitamin A (22–23%), vitamin B-12 (25–28%), and vitamin D (43–50%). However, carbohydrates, fiber, folate, energy, MUFAs, PUFAs, sodium, and total fat increased by <10%, and added sugar did not change with the addition of a serving of milk (Table 4).

**TABLE 4:** Mean intake of nutrients among children aged 2–5 y after addition of 1 cup equal of milk, gender-combined data NHANES 2001–2018.

Nutrient	Age 2 y		Age 3 y		Age 4 y		Age 5 y	
	Baseline	Adjusted	Baseline	Adjusted	Baseline	Adjusted	Baseline	Adjusted
Energy (kcal)	1427 ± 13	1549 ± 13	1515 ± 19	1637 ± 19	1597 ± 19	1719 ± 19	1742 ± 22	1864 ± 22
Carbohydrate (g)	194 ± 2	206 ± 2	207 ± 3	218 ± 3	219 ± 3	231 ± 3	238 ± 3	250 ± 3
Dietary fiber (g)	10.3 ± 0.2	10.3 ± 0.2	11.0 ± 0.2	11.0 ± 0.2	11.7 ± 0.2	11.7 ± 0.2	12.4 ± 0.2	12.4 ± 0.2
Total sugars (g)	104 ± 1	117 ± 1	107 ± 2	120 ± 2	112 ± 2	124 ± 2	121 ± 2	133 ± 2
Added sugars (tsp eq)	10.2 ± 0.2	10.2 ± 0.2	12.2 ± 0.3	12.2 ± 0.3	13.8 ± 0.4	13.8 ± 0.4	16.6 ± 0.4	16.6 ± 0.4
Protein (g)	51.6 ± 0.6	59.7 ± 0.6	53.2 ± 0.7	61.2 ± 0.7	55.0 ± 0.7	63.1 ± 0.7	59.9 ± 1.0	68.0 ± 1.0
Total fat (g)	51.6 ± 0.6	56.4 ± 0.6	55.2 ± 0.9	60.0 ± 0.9	58.1 ± 0.9	62.9 ± 0.9	63.7 ± 1.0	68.6 ± 1.0
Total MUFA (g)	17.8 ± 0.2	19.0 ± 0.2	19.3 ± 0.3	20.6 ± 0.3	20.3 ± 0.3	21.6 ± 0.3	22.6 ± 0.4	23.9 ± 0.4
Total PUFA (g)	9.88 ± 0.17	10.11 ± 0.17	11.1 ± 0.2	11.3 ± 0.2	11.7 ± 0.2	11.9 ± 0.2	12.7 ± 0.2	12.9 ± 0.2
Total SFA (g)	19.3 ± 0.3	22.2 ± 0.3	19.9 ± 0.4	22.8 ± 0.4	21.0 ± 0.4	23.9 ± 0.4	23.0 ± 0.4	25.9 ± 0.4
Calcium (mg)	989 ± 15	1279 ± 15	946 ± 19	1236 ± 19	946 ± 20	1236 ± 20	999 ± 19	1289 ± 19
Magnesium (mg)	193 ± 2	220 ± 2	196 ± 3	223 ± 3	202 ± 3	229 ± 3	212 ± 3	239 ± 3
Potassium (mg)	2007 ± 20	2365 ± 20	1972 ± 29	2330 ± 29	2022 ± 32	2380 ± 32	2077 ± 35	2435 ± 35
Sodium (mg)	2082 ± 24	2185 ± 24	2232 ± 29	2335 ± 29	2397 ± 36	2501 ± 36	2646 ± 44	2750 ± 44
Vitamin A, RE (μg)	556 ± 11	679 ± 11	540 ± 12	664 ± 12	571 ± 15	694 ± 15	572 ± 14	695 ± 14
Folate, DFE (μg)	377 ± 7	389 ± 7	409 ± 9	421 ± 9	443 ± 10	454 ± 10	502 ± 13	513 ± 13
Vitamin B-12 (μg)	4.31 ± 0.09	5.45 ± 0.09	4.08 ± 0.09	5.22 ± 0.09	4.24 ± 0.09	5.37 ± 0.09	4.54 ± 0.11	5.68 ± 0.11
Vitamin D (μg)	6.92 ± 0.13	9.91 ± 0.13	6.13 ± 0.14	9.12 ± 0.14	5.96 ± 0.16	8.95 ± 0.16	6.13 ± 0.18	9.12 ± 0.18

Abbreviations: DFE, dietary folate equivalents; MUFA, monounsaturated fatty acid; NHANES, National Health and Nutrition Examination Survey; PUFA, polyunsaturated fatty acid; RE, retinol equivalent; SEM, standard error of the mean; SFA, saturated fatty acid.

Data presented as least square mean ± SEM after adjusting for gender, ethnicity, and poverty-income-ratio level. Dietary modeling was not performed for children aged 1 y.

### Modeling nutrient intake after isocaloric replacement of caloric beverages at meals with milk

With the isocaloric replacement of caloric beverages with milk (NHANES food code 11100000 milk, nfs) at lunch and dinner (∼0.2–0.4 cups across age groups) among children aged 2–5 y, several nutrients increased significantly (*P* < 0.05) including protein (5–6%), fat (3%), MUFAs (2–3%), PUFAs (1%), SFAs (5–6%), calcium (9–12%), magnesium (3–4%), potassium (3–5%), sodium (1%), vitamin A (8–9%), folate (1%), vitamin B-12 (9–11%) and vitamin D (15–22%); with significant (*P* < 0.05) decreased in carbohydrate (3–4%), fiber (1%), total sugar (5–6%) and added sugar (11–13%) (Table 5).

**TABLE 5:** Mean intake of nutrients among children aged 2–5 y after isocaloric replacement of all caloric beverages during lunch and dinner with milk, gender-combined data NHANES 2001–2018.

Nutrient	Age 2 y		Age 3 y		Age 4 y		Age 5 y	
	Baseline	Adjusted	Baseline	Adjusted	Baseline	Adjusted	Baseline	Adjusted
Energy (kcal)	1427 ± 13	1427 ± 13	1515 ± 19	1515 ± 19	1597 ± 19	1597 ± 19	1742 ± 22	1742 ± 22
Carbohydrate (g)	194 ± 2	187 ± 2∗	207 ± 3	200 ± 3∗	219 ± 3	212 ± 3∗	238 ± 3	229 ± 3∗
Dietary fiber (g)	10.3 ± 0.2	10.2 ± 0.2∗	11.0 ± 0.2	10.9 ± 0.2∗	11.7 ± 0.2	11.6 ± 0.2∗	12.4 ± 0.2	12.3 ± 0.2∗
Total sugars (g)	104 ± 1	99.1 ± 1.3∗	107 ± 2	102 ± 2∗	112 ± 2	106 ± 2∗	121 ± 2	114 ± 2∗
Added sugars (tsp eq)	10.2 ± 0.2	9.03 ± 0.21∗	12.2 ± 0.3	10.8 ± 0.3∗	13.8 ± 0.4	12.1 ± 0.3∗	16.6 ± 0.4	14.5 ± 0.4∗
Protein (g)	51.6 ± 0.6	54.3 ± 0.6∗	53.2 ± 0.7	56.0 ± 0.7∗	55.0 ± 0.7	58.1 ± 0.8∗	59.9 ± 1.0	63.4 ± 1.0∗
Total fat (g)	51.6 ± 0.6	53.2 ± 0.6∗	55.2 ± 0.9	56.9 ± 0.9∗	58.1 ± 0.9	59.9 ± 0.9∗	63.7 ± 1.0	65.8 ± 1.0∗
Total MUFA (g)	17.8 ± 0.2	18.2 ± 0.2∗	19.3 ± 0.3	19.8 ± 0.3∗	20.3 ± 0.3	20.8 ± 0.3∗	22.6 ± 0.4	23.1 ± 0.4∗
Total PUFA (g)	9.9 ± 0.2	9.9 ± 0.2∗	11.1 ± 0.2	11.1 ± 0.2∗	11.7 ± 0.2	11.8 ± 0.2∗	12.7 ± 0.2	12.7 ± 0.2∗
Total SFA (g)	19.3 ± 0.3	20.3 ± 0.3∗	19.9 ± 0.4	21.0 ± 0.4∗	21.0 ± 0.4	22.1 ± 0.4∗	23.0 ± 0.4	24.3 ± 0.4∗
Calcium (mg)	989 ± 15	1078 ± 14∗	946 ± 19	1043 ± 19∗	946 ± 20	1050 ± 21∗	999 ± 19	1117 ± 19∗
Magnesium (mg)	193 ± 2	198 ± 2∗	196 ± 3	202 ± 3∗	202 ± 3	209 ± 3∗	212 ± 3	220 ± 3∗
Potassium (mg)	2007 ± 20	2066 ± 20∗	1972 ± 29	2041 ± 30∗	2022 ± 32	2104 ± 33∗	2077 ± 35	2175 ± 34∗
Sodium (mg)	2082 ± 24	2108 ± 24∗	2232 ± 29	2262 ± 29∗	2397 ± 36	2428 ± 37∗	2646 ± 44	2681 ± 45∗
Vitamin A, RE (μg)	556 ± 11	598 ± 10∗	540 ± 12	585 ± 12∗	571 ± 15	616 ± 15∗	572 ± 14	625 ± 14∗
Folate, DFE (μg)	377 ± 7	379 ± 7∗	409 ± 9	411 ± 9∗	443 ± 10	445 ± 10∗	502 ± 13	504 ± 13∗
Vitamin B-12 (μg)	4.31 ± 0.09	4.71 ± 0.10∗	4.08 ± 0.09	4.51 ± 0.09∗	4.24 ± 0.09	4.68 ± 0.10∗	4.54 ± 0.11	5.04 ± 0.11∗
Vitamin D (μg)	6.92 ± 0.13	7.96 ± 0.13∗	6.13 ± 0.14	7.24 ± 0.15∗	5.96 ± 0.16	7.13 ± 0.17∗	6.13 ± 0.18	7.47 ± 0.17∗

Abbreviations: DFE, dietary folate equivalents; MUFA, monounsaturated fatty acid; NHANES, National Health and Nutrition Examination Survey; PUFA, polyunsaturated fatty acid; RE, retinol equivalent; SEM, standard error of the mean; SFA, saturated fatty acid.Data presented as least square mean ± SEM after adjusting for gender, ethnicity, and poverty-income-ratio level. Dietary modeling was not performed for children aged 1 y.

∗ Different from baseline at *P* < 0.01 by paired t-test.

## Discussion

We used nationally representative NHANES data to understand beverage intake patterns and their relationship to nutrition intake among American children from the ages of 1–5 y of life. The results of the present analysis indicate that with increasing age, the intake of milk, milk substitutes, CB + M, and 100% fruit juice decreased, whereas the intakes of CB, flavored milk, soft drinks, SSBs, and coffee/tea increased. Consequently, the intake of energy and most nutrients from CB + M decreased with age, whereas the intake of fiber and sugars (including added sugar) increased with age. The addition of a serving of milk to the daily diet or isocaloric replacement of all caloric beverages at lunch and dinner with milk resulted in increases in several nutrients to encourage, including calcium, potassium, magnesium, vitamin A, vitamin B-12 and vitamin D, and protein with only a 1–3 g/d increase in saturated fat across both modeling scenarios and a 1–2 teaspoon equal/d (4–8 g/d) decrease in added sugars for replacing caloric beverages at meals.

DGA 2020–2025, American Academy of Pediatrics, and other authoritative bodies have provided dietary recommendations, including for beverages, to promote health and reduce the risk of disease for infants and toddlers [[12], [13], [14], [15], [16]]. Traditionally, the DGAs (prior editions) have only provided dietary recommendations for Americans ages 2 y and older; however, the latest DGA 2020–2025 included recommendations for infants and children from birth to 2 y [13,33]. The average intake of fluid cow milk (including flavored milk) was ∼2.1 cups/d among age 1 y, which aligns with the current daily recommendations of 2–3 cups/d. However, fluid milk intake steadily declined with age, with an average intake of 1.3 cups/d at age 5 y, which is 35% less than the MyPyramid [28] recommended 2 cups/d. Intake of 100% fruit juice also declined by ∼23% from age 1 y to age 5 y, whereas intakes of fruit drinks, soft drinks, and all SSBs increased with age (108%, 395%, and 138%, respectively). Overall, beverage intake patterns are not aligned with current dietary recommendations.

Nutritional adequacy of the diet of infants and toddlers plays an important role in healthy growth and development [1,34]. Inadequate intake of nutrients can negatively affect the development of organ systems and can have lasting consequences [[35], [36], [37]]. Recent data suggest that infants and toddlers consume inadequate amounts of iron, fiber, calcium, vitamin D, vitamin E, and potassium while consuming excess amounts of saturated fat, sodium, and added sugars [13,38]. Because beverages make a significant contribution to dietary intake during early childhood, research shows that what children drink can have a substantial impact on their health. Milk is a nutrient-rich and affordable source of 13 essential nutrients (e.g., high-quality protein, calcium, phosphorus, potassium, zinc, iodine, selenium, magnesium, vitamin A, vitamin D, vitamin B-12, riboflavin, and pantothenic acid) in the United States diet, and is also the leading food source of 3 of the 4 nutrients of public health concern (calcium, vitamin D, and potassium) for children 2–18 y [27,39, 40]. Milk intake among children has been correlated with better diet quality and nutritional adequacy [[41], [42], [43]]. Moreover, a consensus statement on healthy beverage consumption in early childhood, published by key national health and nutrition organizations in the United States, acknowledged the scientific evidence supporting the benefits of milk consumption by young children and recommended 2+ cups of milk for children age 1–5 y [[12], [13], [14], [15], [16]]. Additionally, the statement recommended the avoidance of plant-based milk alternatives (except for fortified soy beverages), flavored and toddler milk, SSB, and beverages containing low-calorie sweeteners or caffeinated beverages [16].

Recognizing the unique nutritional needs and diets of young children (age 2–5 y) are somewhat different from older children and adults, a modeling analysis that focused on the adequacy of the USDA’s food patterns for young children (age 2–5 y) examined a modified dairy group (containing 70% fluid milk as low fat (1% fat) milk) compared with an unmodified dairy group (containing 51% fluid milk as fat-free milk) [44]. The modified dairy group provided more energy (25%), carbohydrate (33%), potassium (26%), vitamin A (19%), folate (22%), vitamin D (36%), and sodium (19%) per cup then the unmodified dairy group. This modified dairy group provided more milk in the dietary pattern, and children were closer to meeting nutrient needs, thus indicating the important role fluid milk has in maintaining nutrient adequacy in this age group. Additionally, dairy and milk intake in early childhood (1–5 y) was positively associated with increased linear growth but not with BMI (in kg/m^2^) or obesity in a recent review of scientific literature [29]. Our data shows that the intake of milk decreases with the growth of infants and toddlers, whereas the intake of SSB increases with age with a consequent decrease in the intake of most nutrients from CB + M. Previously published data also suggest that infants and toddlers consume inadequate amounts of key micronutrients, including iron, fiber, calcium, vitamin D, vitamin E, and potassium, while consuming excess amounts of saturated fat, sodium, and added sugars [13,38]. Our data shows that the intake of milk decreases with increasing age of infants and toddlers, whereas the intake of SSB increases with growth with a consequent decrease in the intake of most nutrients from CB + M. Previously published data also suggest that infants and toddlers consume inadequate amounts of key micronutrients, including iron, fiber, calcium, vitamin D, vitamin E, and potassium, while consuming excess amounts of saturated fat, sodium, and added sugars [13,38].

Despite the universal recommendations from DGA and other authoritative and professional organizations and recognized nutritional benefits, milk consumption continues to decline, and >80% United States population currently consumes less than the recommended intake of dairy [13]. Globally, the consumption of milk and dairy products among children has decreased and a substantial portion of the population fails to meet the recommended intake [45]. From 1977 to 2001, milk consumption in the United States among children decreased by 20% in the number of servings per day (from 3.5 to 2.8 servings/d), ∼11% decrease in the amount per serving (from 460 to 410 mL per serving), with 10% fewer children (from 94% to 84%) consuming milk [45,46]. It is interesting to note that from 1977 to 2001, although the energy intake from milk decreased by 38%, the energy intake from SSB increased by 135%, with a 278 total calorie increase between all age groups [46]. A declining trend in milk intake among children aged 0–5 y was also reported in another analysis of NHANES across 3 decades [47]. Another recent study reported a steady and continuous 41% decline in per capita yearly liquid milk consumption from 1975 to 2017, and the declining trend was attributed to declining consumption frequency [48,49]. A similar declining trend in fluid milk intake was also reported for preadolescent children aged 2–12 y, with ∼30% less consumption (1.7–1.2 cups/d) and twice the percentage of children who did not drink milk on a given day (12–24%) from 1977–1978 to 2007–2008 [49]. Some of the recent decrease in milk consumed as a beverage has also been attributed to increased consumption of plant-based alternative beverages or nondairy alternatives [50], which have grown by >60% since 2013 [51]. Household scanner data from the Economic Research Service confirms that the sale of nondairy alternatives is contributing to the declining sale of fluid milk but also indicates that this may not be the primary driver of these trends [52].

Our data indicates that children aged 1–5 y drink less milk as they age, even though their nutrient needs are increasing. Our earlier analysis of NHANES 2011–2014 also found a decline in total dairy consumption among children after 23 mo of age [53], and in an analysis of NHANES 2001–2018, a similar age-related decline in milk intake was seen as children went from early childhood to their teenage years [54]. Previous cross-sectional and longitudinal studies have reported similar age-related declines in milk intake among children [45]. Decreasing milk and milk substitute consumption with age was concomitant with an increase in consumption of SSB in the present analysis. Previous reports suggest that SSB consumption generally begins during the preschool years and increases with age [21,47], and their consumption is associated with decreased milk consumption, impacting the diet quality and increasing the risk of childhood obesity [55,56]. Higher SSB consumption across eating occasions among 2–4 y old compared to 1–2 y old was also found in an analysis of the feeding infants and toddlers study 2016 [18]. Another cross-sectional study reported increased consumption of SSBs as children grew older, and over 51% consumed SSB on any given day [11], and American infants and children aged 1–5 y did not meet dairy and other food group recommendations and had inadequate intakes of key nutrients such as calcium, vitamin D, and vitamin E [53].

Major strengths of our study were the use of a large, nationally representative, population-based sample of children, which was achieved through combining several sets of NHANES data releases and the use of within-individual dietary modeling. A major limitation of this study is the use of self-reported dietary intake data which have been criticized to be prone to potential reporting bias [57]. Additionally, the results of the dietary modeling are focused on evaluating the maximum effect and, therefore, may not reflect actual individual dietary behavior and can only be used to estimate the potential impact on nutrient intake. However, such modeling offers a relatively easy and reliable technique to evaluate the potential nutritional impact of dietary guidance. Future modeling research should focus on including common plant-based beverages (e.g., soy, almond, oat, coconut, etc.) if and when they become more accepted by children of this age group to better understand if these beverages with their diverse set of nutrients can impact of mealtime beverage choices.

In conclusion, among children aged 1–5 y, the intake of milk decreased, whereas intakes of SSBs, including soft drinks, and added sugar, as well as energy, increased with age, and consequently, the nutrient contribution of CB + M also decreased with age. Additionally, the results of this study show that the addition of a serving of milk or isocaloric replacement of caloric beverages consumed at meals with milk resulted in an increase in protein and several key micronutrients with no change or a decrease in added sugar. The current findings suggest that increased efforts are needed to reverse the decrease in milk intake over time, and as children age, they also provide additional evidence to support specific dietary recommendations for milk.

## Author contributions

The authors’ responsibilities were as follows – KR-J, MAP, CJC: participated in the formulation of the research question, design of the analyses, revision of the manuscript, and the approval of the final version; KF: participated in the formulation of the research question, data analyses, the revision of the manuscript, and the approval of the final version; VLF: participated in the formulation of the research question, design of analyses, NHANES dietary data analysis, interpretation of the data, revision of the manuscript, and the approval of the final version; SA: participated in the interpretation of the data, drafting of the manuscript, revision of the manuscript, and the approval of the final version; and all authors: read and approved the final manuscript.

## Conflict of interest

KR-J, MAP, and CJC are employees of the National Dairy Council. KF and VLF at Nutrition Impact LLC perform consulting and database analyses for various food and beverage companies and related entities, and SA at NutriScience LLC performs consulting for various food and beverage companies and related entities.

## Funding

This work was funded by National Dairy Council.

## Data availability

The datasets analyzed in this study are available in the Center for Disease Control and Prevention repository; available online: http://www.cdc.gov/nchs/nhanes/ [cited 23 August, 2023]. Upon request, authors are willing to help others to replicate or conduct similar analyses.
