# Supplementary material for: Assessment of Milk and Beverage Intake Trends During Preschool Age and Modeling the Nutritional Impact of Replacing Nondairy Caloric Beverages with Milk
Source: Curr Dev Nutr. 2024 Aug 8;8(9):104436. doi: 10.1016/j.cdnut.2024.104436 (PMC11402051; doi:10.1016/j.cdnut.2024.104436)
Supplement: multimedia component 1 [file mmc1.docx]

Assessment of milk and beverage intake trends during preschool age and modeling the nutritional impact of replacing non-dairy caloric beverages with milk.

Author: Kristin Ricklefs-Johnson

**Supplemental Table 1. Nutrient contents of milk (NHANES food code 11100000; milk, nfs) [24]**

| Nutrients | Amount |
| --- | --- |
| Energy | 122 kcal/cup |
| Carbohydrate | 11.7 g/cup |
| Dietary fiber | 0.00 g/cup |
| Total sugars | 12.5 g/cup |
| Added sugars | 0.00 tsp eq/cup |
| Protein | 8.03 g/cup |
| Total fat | 4.83 g/cup |
| Total monounsaturated fatty acid | 1.28 g/cup |
| Total polyunsaturated fatty acid | 0.23 g/cup |
| Total saturated fatty acid | 2.92 g/cup |
| Calcium | 290 mg/cup |
| Magnesium | 27.1 mg/cup |
| Potassium | 358 mg/cup |
| Sodium | 103 mg/cup |
| Vitamin A, RE | 124 µg/cup |
|  |  |
| Folate, DFE | 11.5 µg/cup |
| Vitamin B_12_ | 1.14 µg/cup |
| Vitamin D | 2.99 µg/cup |

DFE, dietary folate equivalents; RE, retinol equivalent
